# Cotton Wastes Functionalized Biomaterials from Micro to Nano: A Cleaner Approach for a Sustainable Environmental Application

**DOI:** 10.3390/polym13071006

**Published:** 2021-03-24

**Authors:** Samsul Rizal, Abdul Khalil H. P. S., Adeleke A. Oyekanmi, Olaiya N. Gideon, Che K. Abdullah, Esam B. Yahya, Tata Alfatah, Fatimah A. Sabaruddin, Azhar A. Rahman

**Affiliations:** 1 Department of Mechanical Engineering, Universitas Syiah Kuala, Banda Aceh 23111, Indonesia; 2 School of Industrial Technology, Universiti Sains Malaysia (USM), Penang 11800, Malaysia; ngolaiya@futa.edu.ng (O.N.G.); ck_abdullah@usm.my (C.K.A.); essam912013@gmail.com (E.B.Y.); tataalfatah83@gmail.com (T.A.); atiyah88@gmail.com (F.A.S.); 3 School of Physics, Universiti Sains Malaysia (USM), Penang 11800, Malaysia; arazhar@usm.my

**Keywords:** cotton wastes, textile, nanomaterials, cellulose nanocrystal, extraction methods, environmental application

## Abstract

The exponential increase in textile cotton wastes generation and the ineffective processing mechanism to mitigate its environmental impact by developing functional materials with unique properties for geotechnical applications, wastewater, packaging, and biomedical engineering have become emerging global concerns among researchers. A comprehensive study of a processed cotton fibres isolation technique and their applications are highlighted in this review. Surface modification of cotton wastes fibre increases the adsorption of dyes and heavy metals removal from wastewater. Cotton wastes fibres have demonstrated high adsorption capacity for the removal of recalcitrant pollutants in wastewater. Cotton wastes fibres have found remarkable application in slope amendments, reinforcement of expansive soils and building materials, and a proven source for isolation of cellulose nanocrystals (CNCs). Several research work on the use of cotton waste for functional application rather than disposal has been done. However, no review study has discussed the potentials of cotton wastes from source (Micro-Nano) to application. This review critically analyses novel isolation techniques of CNC from cotton wastes with an in-depth study of a parameter variation effect on their yield. Different pretreatment techniques and efficiency were discussed. From the analysis, chemical pretreatment is considered the most efficient extraction of CNCs from cotton wastes. The pretreatment strategies can suffer variation in process conditions, resulting in distortion in the extracted cellulose’s crystallinity. Acid hydrolysis using sulfuric acid is the most used extraction process for cotton wastes-based CNC. A combined pretreatment process, such as sonication and hydrolysis, increases the crystallinity of cotton-based CNCs. The improvement of the reinforced matrix interface of textile fibres is required for improved packaging and biomedical applications for the sustainability of cotton-based CNCs.

## 1. Introduction

The demand for textile materials is increasing globally due to population growth and economic development [1]. Textile materials have been widely used in many fields of life [2]. Reference [3] stated that, among world fibre consumption, cotton is the most consumed fibre after polyester. According to global statistics, Reference [4] reported that more than 24 million tons of cotton cloths are produced and used. Textile industry contributes tons of cotton wastes, which affects the environment [5]. As a result of continuous production and utilization of cotton fibres, the wastes generated have proportionately increased over the years, causing significant environmental issues. Reference [6] categorized cotton wastes as pre-consumers and post-consumer wastes. Reference [7] regarded pre-consumer wastes as wastes from processed fibres, including finished yarns, technical textiles, garments off-cuts, nonwovens, and other rejected processing cotton materials. Reference [8] also added that pre-consumer cotton wastes are generated during yarn, fabric, and garment product manufacturing. Reference [9] classified post-consumer wastes as dirty wastes, which include both natural and synthetic materials. Reference [8] further classified post-consumer cotton wastes as discarded wastes at the end of the product’s service life. Reference [10] stated that most of the cotton waste streams are post-consumer. The environmental impact, as a result of the ineffective processing mechanism of cotton wastes, is a major concern. Reference [11] reported that only 15% of cotton garments are recycled in the United States, 19% are incinerated, and 66% are landfilled. The processing and utilization of cotton wastes have the propensity to reduce environmental impact associated with its discharge. In other words, processed cotton wastes can be utilized as adsorbent media for the removal of recalcitrant pollutants from wastewater, as reinforcement materials for packaging and biomedical applications. Furthermore, the various forms of cotton wastes, especially post-consumer wastes, can be utilized as a promising source of cellulose nanocrystals (CNCs) due to its high cellulose contents including its inherent prospects for advanced applications toward environmental sustainability [12]. CNCs are the derivatives of cellulose used for various applications in nanoscience due to its high mechanical strength, high aspect ratio, large surface area, high modulus, and biodegradability [13,14]. CNCs are obtained in the form of rod-like crystalline domain consisting of diameters in the range of 1 to 100 nm and length around 10 to 100 nm [15]. The extent of the crystallinity of CNCs as a functional material depends on the precursor used for the extraction of cellulose and the intensity of the extraction methods. In nanotechnology, the utilization of CNCs as reinforcements for bio-based polymers are attracting remarkable prospects to enhance the properties of biomaterials. Due to its unique properties, many studies have discussed the extraction of CNCs from biomass and have reported the potential applications in varieties of broad-based areas, such as in environmental, packaging, construction, biomedicals, and automobiles [16]. The extraction of CNCs from cotton wastes have remarkable prospects and application in various areas of nanotechnology. In this review, based on current knowledge on the fundamental aspects and properties of cotton wastes, a comparative study of the pretreatment methods for the extraction of CNCs from cotton wastes are comprehensively discussed. The application of cotton wastes as a functional material in different fields of biotechnology for environmental sustainability and health safety have been discussed. This includes the advanced application and future prospects of nanocrystals from cotton wastes. This review will likely increase research interest in cotton wastes as a new source of nanocrystals.

Cotton wastes generated have either been incinerated or landfilled. Few research works on the use of cotton waste for functional application rather than disposal have been done. However, no review has critically discussed research findings and analyses of the state of cotton waste use. In this review, the relevance of our study is benchmarked on three critical aspects including cotton wastes generation and the associated environmental impacts, and functional properties of cotton fibres into functional materials to reduce the environmental impact as a result of disposal and functional biomaterials for sustainable environmental applications. A critical review on this subject is rare. The awareness of functional usage of cotton waste for CNC production has not been fully explored because many studies focus on isolation of CNC from a wood source. Therefore, no review has been written in this respect, which has limited the valorisation of cotton waste for a functional application. This review critically discussed the functional application of cotton waste at micro to nano sizes. This covers cotton fibre and CNC isolation, their application, limitations, and proposed research to combat these limitations. To the best of our knowledge, no review study has discussed the potentials of cotton wastes from source (Micro-Nano) to application. The utilization of the cotton wastes based on its intrinsic functional properties have found wide application in environmental sustainability through dye and heavy metals removal from wastewater as well as geotechnical, packaging, and biomedical applications. The improvement of the reinforced matrix interface of textile fibres is required for improved packaging and biomedical applications for the sustainability of cotton-based CNCs.

## 2. Environmental Impact of Cotton Wastes

Over the last decade, 40% of global fibre production is attributed to cotton [17]. China is the world’s largest producer and consumer of cotton [18]. Reference [19] gave an instance that the Keqiao industrial park, which is one of the largest textile industrial parks in China, has the capacity for more than 3 million tons of varieties of cotton fibres, including 19 billion clothing fabrics. As the global demand for cotton increases, there is an exponential increase in wastes generated. Cotton wastes generated have either been incinerated or landfilled, creating many environmental problems. On a yearly basis, about 15.1 million tons and 1.7 million tons of cotton wastes, are generated in the United States and the United Kingdom, respectively. Reference [20] reported that, in Australia, 76% of cotton wastes were deposited in the landfill in 2016. In recent years, according to Zhang, Wu [19], cotton wastes in China have increased to more than 100 million tons annually, most of which ends in the landfill. Most cotton wastes from the plants add up to the waste streams and are mainly deposited in the landfill without effective management. Reference [17] stated that cotton diseases and pests are disposal issues attributed to cotton wastes discharge. The authors also added that the slow decomposition of cotton in the soil causes cultivation problems. The dyeing of textile products utilizes a lot of energy, water, and chemical bleaching agents. In the process, a high amount of wastewater is generated with harmful effects on the environment [21]. Furthermore, the emission of greenhouse gases during cotton production as a result of the utilization of gaseous fuel contributes to climate change. This includes the potential risks that could be detrimental to freshwater ecosystems. This is because rivers, streams, lakes, and other receiving waters are often the primary receivers of generated wastes during and after the production of textiles materials [22]. The need for sustainable solutions to cotton wastes accumulation is becoming more urgent as world fibre consumption increases. Reference [1] have reviewed by conducting a comparative study and concluded that wastes recycling is a more beneficial and sustainable approach to reduce environmental impact compared to incineration or landfilling. In addition, to reduce the environmental impact as a result of cotton wastes generation, there should be a proper mechanism to process these wastes into functional bio-materials for environmental sustainability. This includes a global waste summit to enact laws, regulations, and legislation with a penal code to promote strict compliance toward creating a circular economy that will minimize the impact of environmental pollution.

### 2.1. Functional Properties of Cotton Fibres

The strength of cotton fibres as a functional material is attributed to its rigidity of a cellulose chain, crystalline structure that allows intramolecular and intermolecular hydrogen bonding [23]. Wang, Farooq [24] stated that cotton fibres have high crystallinity, predominantly composed of cellulose. Reference [25] stated that molecules of cellulose in cotton typically are within 800 and 10,000 units. The strength of cotton fibre depends on the degree of orientation of the molecules in the fibre axis [26]. Reference [24] reported that the tensile strength of cotton fibres is influenced by the degree of orientation of molecular chains and the fibre axis. The authors stated further that the more the molecules are oriented, the higher is the fibre strength. Reference [27] have stated that the breaking strength and modulus of cotton fibres are less than the theoretical value of cellulose crystal due to the molecules shorter than the fibre. The authors revealed that the degree of orientation of the chain molecules and fibre axis determines the extent of its tensile strength for reinforcements. Reference [28] investigated the modulus of elasticity in yarn and revealed that the torsional rigidity of cotton fibre in the yarn is approximately twice as high as a single fibre. According to Reference [29], cotton fibres exhibit a tensile strength of 287–840 MPa, a tensile modulus of 9.4 to 22 GPa, 3–10% elongation, and 7–25% of moisture absorption. The adsorption capacity of cotton fibre depends on the structure of the fibre, the fibre density, immersion, solid-liquid interface, adhesion, and wetting potential [30]. Prakash [31] revealed that cotton fibre has high moisture absorption potential, which makes it better suited for dye application. Reference [32] reported that pure cotton, cotton-hemp blends are inherently suitable as UV absorbers and concluded that naturally pigmented cotton combined with lignin composition in hemp fibres demonstrate better UV absorption capacity. In a related study, Gorade, Chaudhary [33] revealed that cotton fibre is hygroscopic and are non-abrasive with a low mass density, which makes it more suitable for liquid transport applications, such as for medical products. The functional properties of cotton fibre wastes have a remarkable prospect for various applications, such as environmental, packaging, and biomedicine. Reference [34] have reported that cotton wastes contain more than 95% of cellulose. The intrinsic properties of cotton wastes, including its abundance of cellulosic composition, have increased the prospect of its utilization as a precursor for the extraction of nanocrystals.

### 2.2. Applications of Cotton Wastes

**Figure 1:**
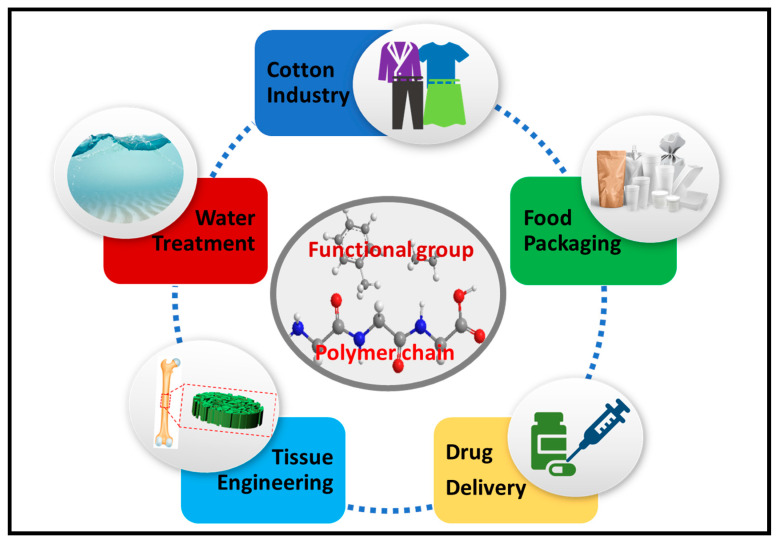
Polymer functionalization and application [35].

During the processing and production of cotton fibres, cotton wastes are generated. Synthesis of these waste fibres have created potentials and a wide application due to their intrinsic properties. The functionalization of polymers for improved surface properties such as adhesion, surface properties including porosity roughness, surface adsorption, and wettability potentials through active functional groups determines its applicability [2,5]. The recycling of wastes into functional materials and application for water treatment, food packaging, biomedical applications, and textiles have been reviewed by Reference [35]. Some functional applications of polymers are illustrated in Figure 1.

The application of cellulose-derived cotton wastes for a wide range of applications, such as environmental including dyes and heavy metal remediation from wastewater, geotechnical, packaging, and biomedical applications, with the potentials of forming composites, is presented in this section.

#### 2.2.1. Environmental Applications

The cost-effectiveness and abundance of cotton wastes can be utilized as the ideal material for the fabrication of highly efficient catalysts and adsorbents for dye and heavy metal removal and for geotechnical applications. For the benefit of the environment, recycling these wastes into value-added products is very significant for environmental sustainability. Çay, Yanık [36] have recycled cotton wastes into biochar to improve the properties of the material. Carbonization of the wastes at a low temperature was applied to improve cotton fabric through the conventional printing process. The produced biochar demonstrated hydrophobic characteristics on the surface of the material, thereby, creating an improved structure with hydrophobic-hydrophilic properties. Cotton waste has been utilized for various applications, such as dyes and heavy metals pollutant removal, due to high sorption mechanisms and synergistic interactions as a suitable adsorption media. The application of cotton wastes for the removal of recalcitrant pollutants from wastewater, soil amendments, and reinforcements are very significant. Cotton fibres is applicable as reinforcement fillers in the matrix for building applications and as a functional material for reinforcement of composites. Cotton fibre properties and techniques of cellulose extraction from cotton waste have been applied for environmental sustainability.

- Water Treatment

Cotton waste is a very significant textile waste that has found application in water purification and wastewater treatment, especially for the removal of emerging and recalcitrant pollutants from wastewater. The removal of pollutants of emerging concerns such as pharmaceuticals, agrochemical personal care products (PPCPs), and dyes have gained interest in recent times. From an environmental perspective, PPCPs are the excreted drugs by humans, sediments, or pollutants from the treatment plant. Meng, Liu [37] have conducted a review study on the potential toxicity of PPCPs. Sathishkumar, Binupriya [38] stated that the disposal of these wastes could be lethargic and hazardous to the environment and the receiving water. However, cotton wastes have been synthesized as adsorbents for the removal of these wastes, which contain organic and inorganic pollutants. Reference [39] stated that the porous nature of cotton wastes determines the adsorption efficiency. Czech, Shirvanimoghaddam [40] studied the adsorption efficiency of carbon microtubes using cotton strips as a precursor for the removal of PPCPs from water. The investigated pollutants from the PPCP were naproxen (NAP), caffeine (CAF), and triclosan (TCS). The efficiency of the carbon microtube achieved 69% of the maximum removal capacity for NAP. In addition, 89.9% removal of TCS and 98% removal of CAF from PPCP was achieved. The removal of recalcitrant pollutants, such as dyes and heavy metals, using cotton wastes have been reported for dye and heavy metal removal.

- Dye Removal

**Figure 2:**
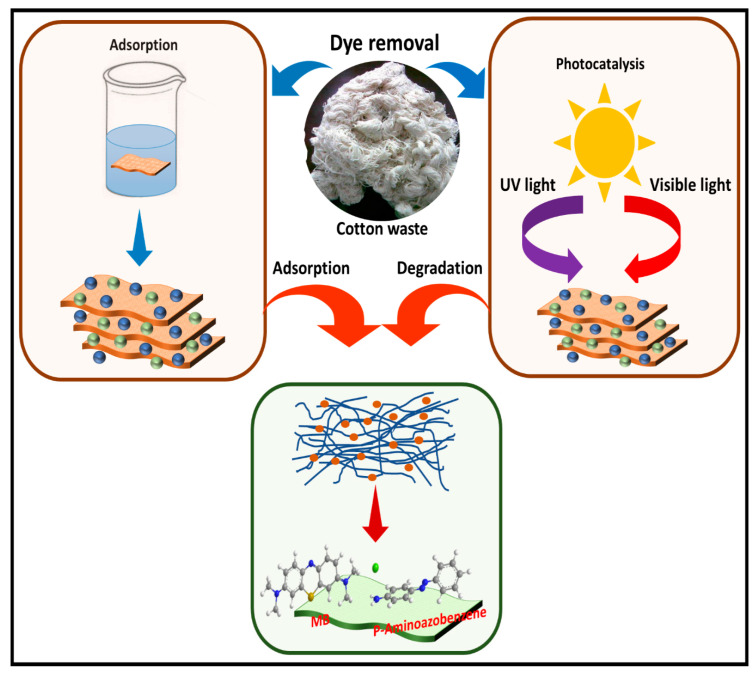
Adsorption and photocatalysis of dye removal using a waste cotton cloth fibre composite.

The application of cotton wastes for the adsorption of synthetic dyes is economically viable because of the availability and ease of processing the wastes. Cotton wastes have been widely applied for the treatment of cationic dyes [41,42]. Qin, Guo [43] have reviewed methods of removal of dyes from aqueous solution. Some of the methods including Fenton oxidation, advanced oxidation, and ozonation have been applied widely in wastewater treatment. A treatment system using adsorption and a photocatalytic process on waste cotton composite film for the removal of dye is illustrated in Figure 2. Qiu, Shu [44] reported that, in a photocatalytic process, TiO_2_ has good photocurrent activity and chemical stability. Although the light absorption of TiO_2_ is limited to ultraviolet light, the energy of ultraviolet light accounts for about 4% of solar energy, and, therefore, restricts its practical application. A schematic illustration of adsorption and a photocatalytic process of pollutant removal in solution.

The efficiency of the processed cotton wastes for the removal of different forms of dyes with a focus on the adsorption method is presented in this review due to its low cost of treatment and high removal capacity from the solution. Table 1 shows the most recent application of cotton wastes for dye adsorption. Song, Li [45] investigated the adsorptive capacity of poly acrylethyl trimethyl ammonium chloride cotton (PA-cotton) for the removal of reactive scarlet 3BS dye. The result indicated that an increase in dosage and temperature favoured the adsorbent, which signified the applicability of the modified cotton wastes for dye removal. Maximum adsorption capacity (Qmax) from the Langmuir isotherm model for the PA-cotton was 540.54 mg/g, which proved to be higher than the widely applied activated carbon and unmodified cotton by 292.18 and 2702.70 times, respectively. The significance of increase in temperature and dosage improved on the adsorption capacity and regeneration potential of the cotton wastes for reuse. Sivarajasekar, Baskar [46] also indicated the prospect of cotton wastes for dye adsorption. The effect of chemisorption influenced maximum adsorption uptake of basic red 2 (BR2) and basic violet 3 (BR3), even though the decrease in adsorption efficiency may likely occur. The increase of initial dye concentrations may likely inhibit the uptake of dyes due to the boundary layer resistance of active pores on the surface of the waste cotton seed. The decrease in the adsorption capacity may also occur due to the unavailability of active sites and pores on the waste cotton seed particles for the attachment of solutes of the dye molecules. Haque, Remadevi [47] investigated the adsorptive capacity of cotton gin trash for the uptake of methylene blue. The result was compared with the uptake of methylene blue onto polypyrole-coated cotton fabrics [48]. Higher sorption capacity was achieved on the polypyrole-coated cotton fabrics as against cotton gin wastes. Comparatively, cotton gin wastes demonstrated lower uptake of methylene blue (MB). This is because the sorption of methylene blue on cotton gin wastes had less attraction toward the cellulosic surface due to the anionic nature of the dye. Tunc, Tanacı [49] used cotton stalk and cotton hull for the adsorption of Ramazol Black B from an aqueous solution.

The investigation revealed that higher sorption capacity was observed on the cotton hull adsorbent than cotton stalk even though the adsorption efficiency for both adsorbents was not very high. The pH of the adsorbate affected the aqueous chemistry and the binding surface of the adsorbents. It was found that cotton wastes, which are predominantly natural cellulosic fibres, are negatively charged as a result of the presence of hydroxyl groups in cellulose. The higher uptakes may likely be obtained at a very acidic pH due to the electrostatic interaction between the positively charged surface of the adsorbent and the negative charge of the Ramazol black B dye anions. Furthermore, Deng, Lu [50] reported that cotton stalk demonstrated less sorption capacity for methylene blue, but the effect of modified sulphuric acid-treated cotton stalk achieved very high adsorption efficiency, which is similar to phosphorus-treated cotton stalk. Cotton wastes bio-adsorbents are potentially suitable for the remediation of dyes from aqueous solutions. Cotton wastes adsorbents have demonstrated promising potentials for the removal of hazardous wastes. However, desorption of dyes on the spent adsorbents without compromising the quality of air safety through incineration requires a sound and sustainable technological approach. This requires intensive research.

**Table 1:** Adsorption efficiency of textile wastes for dyes.

Precursor	Adsorbate	Qmax (mg/g)	References
Cotton wastes	Methylene blue solution	76.0	[47]
Cotton waste	Aqueous solution of Alizarin red dye	73.8	[51]
Cotton wastes	Dissolution of Bi(NO_3_)_3_5H_2_O in a solution of Rhodamine B and Methylene Blue	93.7 97.04	[43]
Cotton fibre	Aqueous solution of Methylene blue dye	95.6	[48]
Cotton stalk	Aqueous solution of Methylene blue	147.06	[50]
Sulphuric acid-treated cotton stalk	Aqueous solution of Methylene blue	555.56	[50]
Phosphorus acid-treated cotton stalk	Aqueous solution of Methylene blue	222.22	[50]
Cotton waste fabrics	Methylene blue and Rhodamine 6G (R6G) solution	185.63 118.21	[52]
Waste cottonseed	Basic red 2 (BR2) and solutions Basic violet 3 (BV3)	50.11 66.69	[46]
Cotton fibre	Concentration of crystal red and methylene blue	175.1 113.1	[53]
cotton hull waste CNC from cotton waste	Reactive blue, red concentration Methylene blue solution	12.91	[54]

- Heavy metals removal

Heavy metal ions are persistent environmental pollutants. They are toxic and hazardous at a low concentration [55]. Heavy metals constitute environmental problems when they exceed the required threshold. The precipitation of metal ions on the surface of activated carbon is the conventional method of metal ion sequestration, while being quite expensive. To obtain cheaper adsorbents, the synthesis and application of cotton wastes for heavy metals removal from wastewater have been studied [56,57,58,59]. Table 2 illustrates the most recent studies of heavy metals adsorption on cotton wastes as an adsorbent media. Mihajlović, Vukčević [58] investigated the sorption capacity of waste cotton yarn for the removal of heavy metal ions from an aqueous solution. High removal of Pb ions was achieved on waste cotton yarn in a binary mixture, unlike in a single solution. This is because the influence of other ions species in the adsorbate favourably induced the removal of Cr. Although, slight removal of Pb ions was achieved. The sorption of Cd in the binary mixture was not affected by the presence of Cr, which implies that there was no competitive interaction of ions in the binary mixture. Despite the high uptake capacity in the binary mixture, there was low removal efficiency of the adsorbent for arsenic removal. This problem is caused by the repulsion of a negatively charged surface and negative ion surface. Another problem is that ions bind on different active sites. This may likely cause a different mechanism of adsorption.

Ma, Liu [60] studied the adsorption of Cd, Cu, Pb, Fe, and Zn onto waste cotton fabrics. The results indicated a high adsorption efficiency of the adsorbent for the investigated heavy metals. The result revealed that the adsorption of all the metal ions increases as the dosage grows. For the metal ions to be effectively removed from the industrial effluent, the treatment volume should be under control below 1932 mL. To achieve this benchmark in this work, 420-min bed contact time was achieved to process 1932 mL effluent. Yahya, Yohanna [56] reported an adsorption capacity of 27.65 mg/g and suggested that the monolayer surface influenced the removal of Pb (II) onto the cotton hull. Mendoza-Castillo et al. (2014) reported the sorption of Pb (II), Cd (II), Zn (II), and As (II) and attributed the high adsorption efficiency to the active functional groups and complex compounds when compared with other studies. The presence of Pb (II) on the surface of the denim waste influenced the removal of arsenic (II).

**Table 2:** Adsorption of heavy metals on textile wastes.

Precursor	Adsorbate	Qmax (mg/g)	Reference
Waste cotton Yarn	Aqueous solution of Pb, Cd, Cr, As ions	890.8 191.7 236.2 72.90	[58]
Waste cotton fibres	Inorganic pollutants As (III) Inorganic arsenic Arsanilic acid (ASA) C_6_H_8_AsNO_3_ Roxarsone (Rox C_6_H_6_AsNO_6_)	126.5 164 261.4 427.5	[61]
Char-FeCl_3_ from cotton waste Char-FeCl_2_ from cotton waste Char-FeCit from cotton waste	Cr(VI) solution, HNO_3_, NaOH Concentration	73.79 68.87 43.84	[57]
Cotton hull	Pb(II) from gold mining liquid effluent	27.65	[56]
Cellulose from waste cotton fabrics	Heavy metal nitrate salts (CuNO_3_)_2_, Cd(NO_3_)_2_, and Pb(NO_3_)_2_ Cd, Cu, Pb, Fe, and Zn investigated	Cd: 99.8 Cu: 99.8 Pb: 99.7 Fe: 98.0 Zn: 99.9	[60]
Cotton fibre	Aqueous solution of Cu(II) ions Aqueous solution of Pb(II) ions Aqueous solution of Cr(III) ions	81.97 123.46 72.99	[59]
Denim fibre scraps	Aqueous solution of Pb(II) Aqueous solution of Cd(II) Aqueous solution of Zn(II) Aqueous solution of As(II)	9.83 2.71 2.69 1.23	[62]
Cotton fibre	Heavy metal solution of Cu(II), Zn(II), Pb(II), and Cd(II)	6.12 4.53 8.22 21.62	[63]
Cotton fibre	Aqueous solution of Cu(II) and Pb(II)	88.9 70.6	[53]

This synergistic interaction implies that competitive adsorption better described the sorption mechanism. Reference [59] investigated the use of cotton fibre for the sorption of Cu (II), Pb (II), and Cr (III) and attributed the sorption capacity of the adsorbent to the effect of increase in pH, contact time, and initial concentration of the adsorbate. The result from the sorption capacity of cotton wastes from the most recent studies indicated the effectiveness of the wastes as an alternative to commercial activated carbon for the adsorption of heavy metals from aqueous solution. The application of cotton wastes for the removal of metal ions has the prospect of ensuring safety for both the environment and the receiving water.

The removal of dyes and heavy metals depends on the chemical composition of the analyte and the nature of the wastes. Spent cotton adsorbents contain toxic substances, which have a severe environmental impact. Desorption of these hazardous wastes is required to ensure environmental sustainability. At the end of the adsorption of dyes on cotton wastes, not all reactive dyes are adsorbed. Consequently, the dyeing baths could generate enormous hazardous effluents.

Hu, Shang [64] have studied the use of catalytic ozonation for effluent reuse and reported that catalytic ozonation could regenerate cotton effluent and can be reused twice without compromising fabric quality. In order to regenerate spent cotton wastes for dyes and heavy metals removal, Yousef, Tatariants [65] have reported that leaching treatment is favourable when compared to incineration, which may further constitute a pollution effect. The authors carried out a leaching test for the removal of dyes from cotton wastes using nitric acid leaching and regenerated the used acid by activated carbon. This technique could recover used cotton fabrics by desorption including the spent acid. The desorption of dyes was attributed to the significance of sound waves during leaching, which influenced breaking down of bonds held by a polar interaction or Van-der Waals forces. This implies that the reduction of carbon footprints is achievable by recycling million tons of cotton wastes that could end up in the landfills and incinerators. Surface modification of highly efficient cotton wastes precursors have been effectively applied for wastewater treatment. Challenges such as surface modification of cotton fibres derived CNC for improved adsorption capacity using various pre-treatment methods have not been researched.

- Geotechnical applications

**Figure 3:**
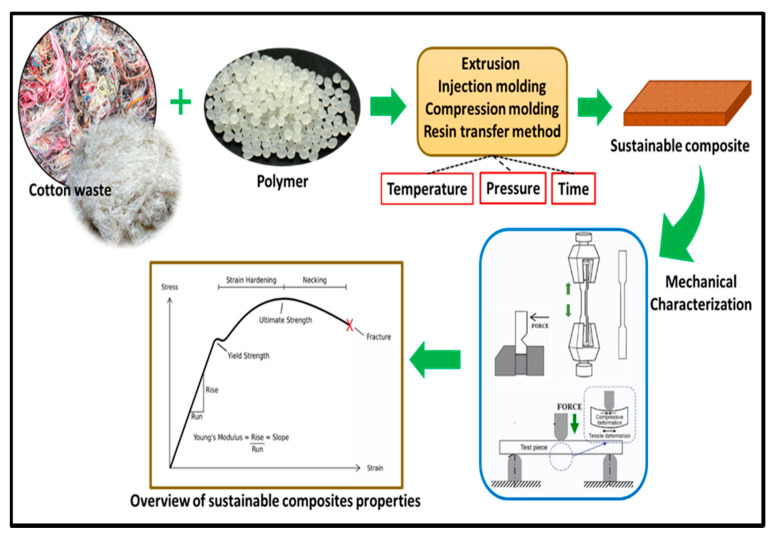
Sustainable composite properties for effective application [69].

Processing of cotton waste fibres for the reinforcement is of huge environmental and economic benefits. For building applications, the processing of cotton fibres to form fibre-reinforced composites has been reported by Reference [66]. Reference [67] combined cotton wastes and limestone powder wastes for the production of low-cost, lightweight composite for building applications. The new material exhibited high energy absorption capacity and mechanical properties compared to the conventional concrete bricks. In another study, Reference [68] utilized cotton fibres for the reinforcement of composite sheets for a building application while polyester resin served as the matrix. The ultimate tensile strength and flexural strength increases as the reinforced cotton fibre filler was increased in the composite. This suggests that the incorporation of cotton fibre in a matrix improved the mechanical properties of the composite. The properties of the prepared bio-composites are very significant for the insulation of building applications. A schematic representation of sustainable composite properties for effective application of the cotton fibre composite is illustrated in Figure 3.

Holt, Chow [70] developed bio-composites from cotton carpel blended with Kenaf, flax, cotton stalks, and southern yellow pine and revealed that composite blends with cotton carpel demonstrated improved mechanical properties when compared to an unblended composite. Authors attributed the behaviour of the cotton carpel to its low modulus of rapture. The strength properties of the bio-composite depends on the physical and mechanical properties of the cotton fibers as filler, the fiber orientation, and the manner the components were combined in the composite structure. Reference [71] reinforced the geopolymer composite using cotton fabrics via a lay-up technique. An increase of cotton fibre further improved the flexural strength of the composite. The authors attributed the improvement of the flexural strength to the increase in the number of woven fibres in the composite. It can be suggested that the lower layer of the cotton fabrics in the composite resisted shear failure and sustains the applied load to the composite. Reference [72] reported the reinforcement of fly ash-based geopolymer using cotton fibre. The porosity of the geopolymer increased as the percentage weight of cotton fibre increased in the composite. The improved flexural strength of the composite could be attributed to the good adhesion of the cotton fibre in the matrix. This could be as a result of the good dispersion of the cotton fibres in the matrix. Improvement of geosynthetic materials using recycled cotton wastes have found application as filler for reinforced structures. Reference [73] investigated the effect of mechanical properties of cotton fibre as filler in polyvinyl chloride (PVC) composites under quasi-loading. The increase in stiffness of the composite is attributed to the increase in the filler, suggesting improved mechanical properties. Reference [74] reported that biopolymers have found useful applications in soil amendment. The strength of cotton fibre-reinforced soil was found to increase compared with unreinforced soil [75]. Sadrolodabaeea, Claramunt [76] fabricated composite materials from short fibre textile materials from cotton wastes for building insulations. The compressive strength, stiffness, and durability properties were found to enhance the composite. This can be attributed to the post cracking performance of the cotton waste composite due to the compressive strength and flexural stiffness, which indicated an improvement in energy absorption and suitability as building components.

In another study, Muthuraj, Lacoste [77] reported the incorporation of cotton fibres for the improvement of the properties of the polylactic acid (PLA) composite. Improved mechanical properties of the composite can be attributed to the addition of fibre, which enhanced the crystallization ability of the PLA in the composite. Improved mechanical properties can be achieved using cotton fibre as a filler in composite applications.

#### 2.2.2. Packaging Applications

**Figure 4:**
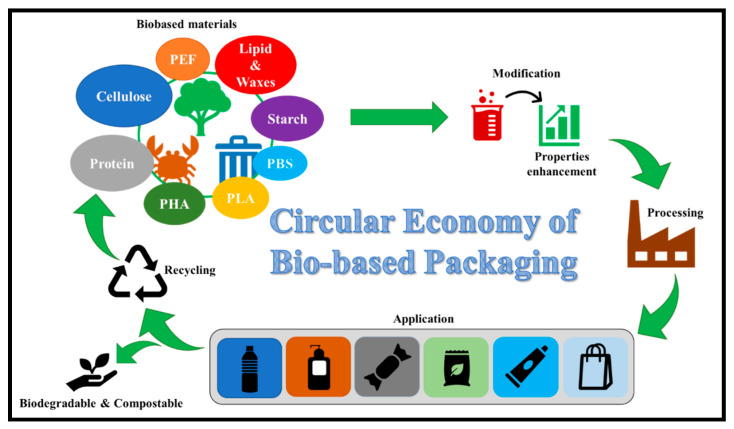
Schematic of bio-derived materials for packaging applications [79]. (PEF: polyethylene furanoate, PLA: polylactic acid, PBS: polybutylene succinate, PHA: polyhydrodyalkanoate).

Technology and engineering sectors are key drivers of innovative waste management strategies to ensure environmental sustainability. Waste engineering and an effective technological approach may well create waste management solutions through recycling of industrial wastes, especially cotton wastes, which are the most generated textile wastes. The concept of bioeconomy has created a sustainable approach to processing cotton wastes into bio-products. Leal Filho, Saari [78] have reported that effective circular design limits the utilization of additives and chemicals for cotton wastes recycling for packaging materials. Reichert, Bugnicourt [79] have reviewed the potentials and significance of processing cellulose from biobased materials for bio-packaging applications. The concept of a bioeconomy can be derived from cotton wastes processing for sustainable packaging application, as illustrated in Figure 4. Circular design toward bio-packaging using cotton wastes as a functional material has a promising prospect.

At a structural level, cellulose derivatives from bio-cellulose composites are an attractive prospect for sheet coating and binders [12]. Fibres of cellulosic composition have a high degree of polymerization, including high modulus and tensile strength for the selection and fabrication of bio-composites. According to Reddy [80], cotton-derived polymer composites can be blended with fillers and additives. After then, they can be reprocessed into different forms. Ramamoorthy, Skrifvars [81] reported that handspun yarns could be made from cotton wastes blended with fibres of feathers for packaging applications. Montava-Jordà, Torres-Giner [82] stated that cotton fibres with a diameter in the range of 10–30 µm and a high aspect ratio improved the mechanical properties in a polymer composite for non-food packaging applications. High aspect ratio cotton fibers in composite reinforcement provides high tensile strength, which implies that cotton fibre reinforcement in a composite have good mechanical properties suitable for packaging applications.

The processing of cotton fibres provides an interesting phase in bio-composite applications due to their elasticity and high tensile strength. The application of bio-composites has focused on synthetic thermoplastics or thermosets and other non-biodegradable polymers as a matrix. Cotton fibres have been applied with other fibres to form bio-composites of good thermal and mechanical properties. Reference [83] have incorporated cotton waste-derived CNF as a reinforcement of PLA/chitin bio-composite and suggested that the improved mechanical, thermal, and wettability property of the bio-composite was attributed to the addition of the filler in the bio-composite.

Meekum and Kingchang [84] demonstrated that the PLA bio-composite with cotton fibre and empty fruit bunch (EFB) from oil palm showed high biodegradability, even though there was a reduction in the flowability of the material when the cotton fraction was increased. This challenge can be eliminated by injecting reactive plasticizers into the bio-composite. de Oliveira, de Macedo [85] have reported that composites in which cotton fibres were used as fillers demonstrated effective packaging potentials when compared to PLA or thermoplastic starch. The authors concluded from the result that the incorporation of 20% cotton fibre demonstrated better mechanical properties when compared to PLA. Furthermore, Montava-Jordà, Torres-Giner [82] have revealed that the injection of recycled cotton fibre improved the thermal and mechanical strength of a bio-poly (ethylene terephthalate) (bio-PET) biopolymer composite for food packaging applications. Hou, Sun [86] have conducted a comparative study of cotton fibre blended with polypropylene fibres by compression moulding under optimized conditions. The cotton stalks were treated using alkali treatment by steam flash explosion and a combination of both methods. The authors concluded that the polypropylene-reinforced composite with the cotton stalk by a combined alkali-steam explosion exhibited excellent mechanical properties including stability in water due to the large surface area attributed to the highest cellulose content obtained from the combined process. Cotton wastes have demonstrated inherent properties that favour its utilization as a packaging material.

#### 2.2.3. Biomedical Applications

**Figure 5:**
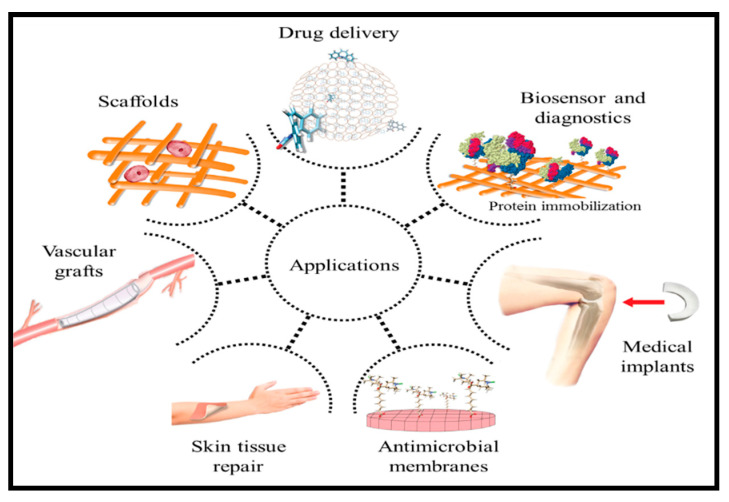
Biomedical application of cellulose-based materials [88].

Biopolymers are often processed for bio-medical packaging, anti-microbial materials, biosensing, and tissue engineered applications [87]. Cotton is a typical example of natural fibre, which contains more than 95% cellulose and have been widely used for bio-medical packaging, anti-microbial materials, biosensing, and tissue engineered applications. Abdul Khalil, Adnan [88] reviewed the biomedical application for cellulose nanofiber and revealed numerous potentials for cellulose nanofiber materials. This includes drug delivery, biosensing, antibacterial, medical implant, tissue engineering, and so on (Figure 5).

- Antimicrobial Materials

**Figure 6:**
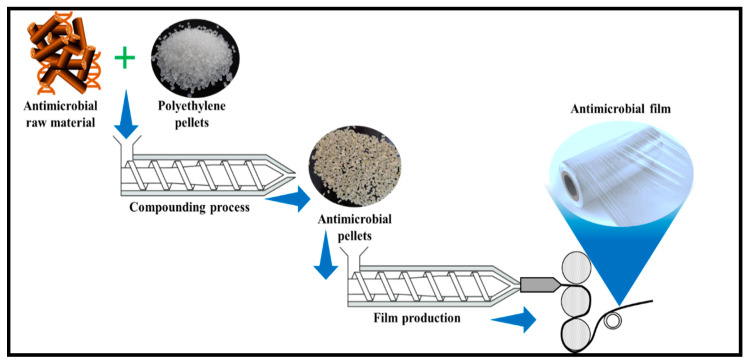
Production of antimicrobial film from antimicrobial raw material.

The unique physicochemical characteristics of cotton fibres are attributed to the available highly reactive functional groups, such as the hydroxyl groups on the surface of the cellulose-based material, which have the tendency to react with other functional groups such as carboxylic acids amines, and aldehydes, resulting in diverse properties. Hence, the prospect of producing antimicrobial biofilm can be achieved for biomedical packaging applications [89]. The production of antimicrobial films from antimicrobial raw materials is shown in Figure 6.

Tavakolian, Jafari [90] have reported that, through surface modification, cellulose material creates a wide range of compounds that can be grafted on its structure. Such compounds include proteins, antibiotics, and nanoparticles. The prevention of cross-infection and cross-contamination have been effective by incorporating antimicrobial agents into fabric materials. Vignesh, Suriyaraj [91] have produced a membrane from cotton micro-dust wastes via thermochemical treatment and a spin coating method. The authors reported that the membrane demonstrated good tensile and rheological properties. The membrane was effective for the absorption of antibiotics and antimicrobial activities against *E. coli*. Coradi, Zanetti [92] have synthesized cotton fabrics as a precursor for the production of antimicrobial textiles. The investigation of antimicrobial activity was carried out using strains of gram-positive bacteria staphylococcus aureus and staphylococcus epidermis, strains of gram-negative bacteria escherichia coli and pseudomonas aeruginosa, and yeast strain of candida Albicans. The enzymatic immobilization and characterization were conducted using pre-bleached cotton fabrics. The immobilization assays indicated that there was enzymatic immobilization on the modified cotton fabrics. It was suggested that oxidation of cotton fabrics via periodate reaction could be an effective alternative to functionalization and immobilization of the investigated enzymes. Zhang, Yang [93] reported that finished cotton fabrics demonstrated excellent bacterial and anti-fouling properties without affecting their water and air permeability when compared to raw cotton. The intrinsic behaviour was attributed to their breaking strength, tearing strength, bursting strength, and hydrophilic behaviour. Vinod, Sanjay [94] stated that, when natural fibres are used as reinforcements in a composite, the hydrophilic behaviour of the fibres and the hydrophobicity of the matrix are the major challenge. Dassanayake, Wansapura [95] fabricated a composite membrane by incorporating cellulose-cadmium (Cd)-tellurium (TE) quantum dots on a cellulose matrix obtained from a waste cotton linter and investigated the antibacterial activity of the composite using staphylococcus aureus. The in vitro analysis revealed that the composite membrane inhibited biofilm formation [96]. The synthesis of chitosan-silver nanoparticles on cotton fibre was reported to achieve 100% antibacterial activity against Gram-positive and Gram-negative bacteria [97]. Wang, Yin [98] evaluated the antimicrobial efficiency of synthesized and grafted polymeric N-halamine precursors (PSPH) onto cotton fabrics by investigating the hemostatic behaviour and the adhesion characteristics of platelets and red blood cells using the modified mesoporous cotton-based materials.

The authors revealed that the bioactive behaviour of the mesoporous surface has the potentials for attaching red blood cells to the surface of the materials. The modified N-halamine polymer was found to have a higher surface area and pore volume, resulting in the increase of a blood clot index. The synthesis of the membrane from cotton wastes is potential candidates for biomaterial applications, such as coating. The incorporated, extracted cellulose from the cotton into membranes have prospects for biomedical packaging, wound dressing applications, and drug delivery systems.

- Biosensing and Diagnostic

Cotton fibres have promising potentials for the integration of biosensors [99]. Idumah [100] have reported that the integration of sensors within cotton fibres has improved physicochemical properties such as flame resistance, UV protection, and antimicrobial behaviour. In view of fabricating a biosensor from cotton, Subbiah, Mani [101] have developed a biosensor from nanostructured ZnO-modified cotton fabrics by modifying the surface of the carbon-cellulose fabric via a sol-gel technique toward enhanced ultraviolet protection. It was reported that the modified functionalized cotton fabric exhibited maximum UV protection. Authors attributed it to the scattering effect within the layers of ZnO, which influenced the enhanced absorption of UV light. Khattab, Fouda [102] have produced a biosensor from cotton gauze by creating a thin layer of alginate via a dip-coating method.

**Figure 7:**
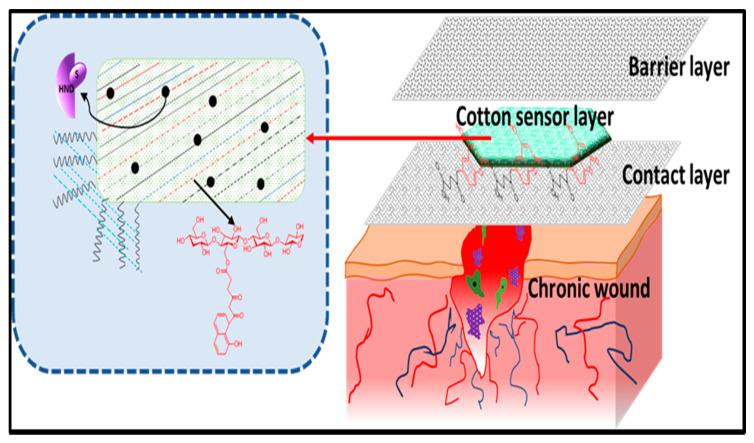
Cotton-based biosensor for wound dressing [103].

The obtained cotton sensor was co-encapsulated in a biopolymer shell by loading the sensor on a pH probe. The sensor system monitored the enzymatic reaction of urease through a pH-responsive tricyanofuran hydrazone chromophore. Under the atmospheric conditions, the encapsulated sensor recorded a detection limit in the range of 0.1 ppm and 250 ppm. The findings revealed that cotton gauze strips that coated the sensor system can be applied for the implantation of a smart bandage. A cotton-derived biosensor was applied for wound dressing. Reference [103] fabricated a cotton-based protease sensor and was applied for chronic wound dressings. A schematic representation of the fabricated sensor is presented in Figure 7.

Abdelrahman, Fouda [104] have developed a colourimetric swab using a molecular switching hydrazone probe in calcium alginate by encapsulating the probe immobilized onto cotton. The micro-encapsulated cotton probe exhibited a colour shift from light yellow to purple. The result of the morphology study of the colourimetric sensor on a pad of dry cotton fibre indicated the colour-fastness and air permeability. Hence, the sensor can be applied for real-time identification of the status of sweat fluid and for other real-time drug test assessments. Song, Xu [105] produced a flexible sensor from polyurethane silver nanowires on cotton yarn via a dip-coating technique. The pressure sensor could effectively be tuned by changing the amount of polyurethane and silver nanowires. The pressure sensor was reported by authors to be conductive, durable, highly sensitive, and stable. It indicated that a conductive pressure sensor with properties such as a low detection limit, working stability, and high sensitivity have the potential to distinguish signals and frequencies and is applicable for real time assessments.

- Tissue Engineering

**Figure 8:**
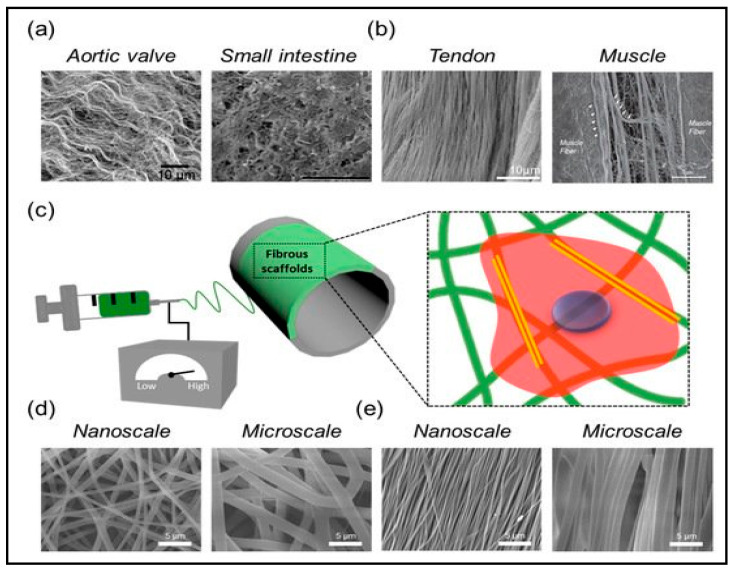
Pore structures of the tissue-engineered scaffold from fibre (**a**) aortic valve & small intestine (**b**) tendon & muscle (**c**) fibrous scaffold (**d**) nanoscale & microscale (**e**) nanoscale & microscale [107].

Cotton fibres have been explored for wide applications in tissue engineering through the regeneration of tissues from cell growth supported by biomaterials. Cotton materials have been explored for the fabrication of scaffolds for tissue-engineered applications. King, Chen [106] reported that woven fabrics have effective mechanical strength and structural stability due to properties such as porosity and thickness. A porous structure of a tissue-engineered scaffold derived for fibre is shown in Figure 8.

Singh, Dutt [108] stated that cotton cellulose exhibits functional material properties for scaffold designs. The authors fabricated the scaffold using cotton microfiber, and they investigated the mechanical and physicochemical properties, including the osteogenic properties of the derived cotton scaffold, by crosslinking with citric acid and modification thereafter with gelatin. The porosity and swelling tests revealed the hydrophilicity of the scaffold and also its nontoxicity, as observed in the micrograph. The authors attributed the water absorption potential of the scaffold to the hydrophilic functional groups [109]. Xu, Wu [110] stated that the hydrophilic properties of scaffold influence the interaction between the materials and the cells. The increase in the hydrophilic property of the citric acid crosslinked gelatin scaffold influenced the rate of biodegradability and the adhesion property of the cell in vivo. Nelson, Tallia [111] have developed electrospun, cotton, wool-like silica/gelatin with covalent coupling applicable as a regenerative medicine scaffold. The investigation included the effect of electrospinning process variables of sol viscosity (and ageing time) and amount of coupling agent on the 3D morphology of the fibres, including their structure and dissolution. Authors attributed the 3D structure of the cotton wool fibre to a slow solvent evaporation rate as a result of high humidity and the elongation of the fibres to form optimal sol viscosity. The observable less congruence in the dissolution of silica and gelatin could be a result of the higher surface area of the spun fibres attributed to the evolution of the hybrid structure. The breaking down of the silica and the crosslinking and release of the gelatin molecules significantly influenced the application of 3D fibres as scaffolds. Liu, Liao [112] synthesized a nanofiber core-spun yarn with nylon filament as core and poly (L-lactide-co-caprolactone) (PLCL) nanofibers as the shell via core-spun electrospinning technology. This was designed to obtain a nanofiber vascular scaffold. The nanofibers of core-spun yarn nanofiber vascular scaffolds demonstrated a directional orientation when compared to conventional nanofiber vascular scaffolds. This indicated that core-spun yarn nanofiber vascular scaffolds have the potential to overcome the weakness of the cohesive force between nanofibers while maintaining effective mechanical properties in the axial and radial directions. The applications of nanomaterials create promising applications in biological and bio-medicine for health safety. The use of nanoparticles via surface modification of cotton wastes with multifunctional properties has promising potential in nanotechnology. The functional properties of cotton fibre can further be explored on a nanoscale for the extraction of CNC.

## 3. Extraction of CNC from Cotton Wastes and Applications

**Figure 9:**
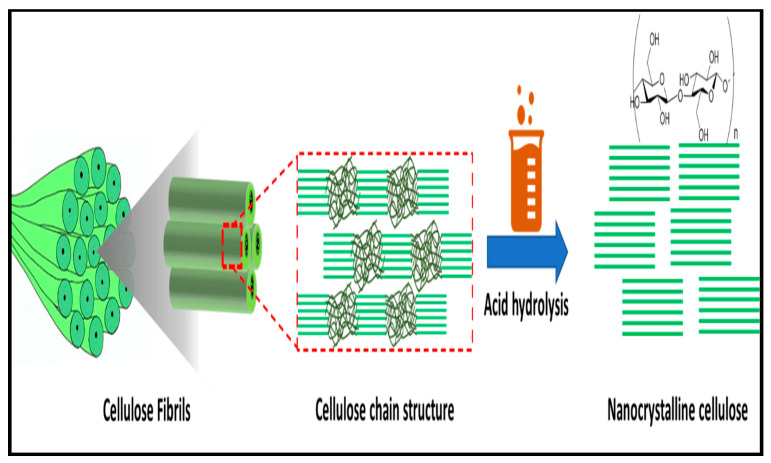
Schematic of nanocrystalline cellulose extraction from cellulose chain using acid hydrolysis for the removal of the amorphous region [115].

The conversion of cotton wastes into valuable nanomaterials is attracting research attention. From an environmental point-of-view, the recycling of cotton wastes, which is the most generated textile waste, is very significant for the protection of the ecosystem. Cotton fibre is an abundant renewable resource. Therefore, cotton waste remains a suitable candidate for the extraction of cellulose and a precursor for the production of CNC. Cotton fibres consist of three major components, namely, cellulose, hemicellulose, and lignin [113]. The crystalline region contains an abundance of cellulose content. The effect of bleaching could further increase the cellulose composition in cotton fibre [114]. The hemicellulose and lignin are the other components of cotton fibre and are amorphous in nature [3]. A schematic illustrating extraction of CNC from a cellulosic precursor is illustrated in Figure 9.

However, the abundance of cellulose composition in cotton waste has increased the prospect of its utilization for the production of CNC. Due to its inherent properties such as high strength, large surface area, high modulus, unique optical properties, and biodegradability potentials, several extraction methods have been explored for the production of CNC from cotton wastes. The degree and extent of the crystallinity of the isolated CNC from cotton wastes depend on the pretreatment methods and the processing conditions utilized. CNC is often good biodegradable reinforcement materials and has become a focus of attention for producing biodegradable composites due to their excellent mechanical properties. However, an effective processing mechanism of cotton wastes into functional materials for the extraction of CNC is rarely reported. Isolation of CNC from cotton wastes have been rarely explored. Much focus is on wood-based plants for commercialization purposes. The prospect of cotton-derived CNC has been explored as reinforcement material to improve the functional properties of composite films for packaging applications [116]. Similarly, the advanced applications of cotton-based CNC have been reported for improved biomedical applications [117].

### 3.1. Chronological Study of Cellulose Extraction from Cotton Wastes

Cotton wastes have been explored as a suitable precursor for the extraction of cellulose. In the early years of textile production, the extraction of cellulose from cotton wastes was achieved by the removal of nanocellulose (NC) compounds. A significant evolutionary trend has been achieved over the years for the extraction of cellulose from cotton wastes. In earlier studies, several pretreatment methods have been used for the extraction, such as chemical treatment using sulphuric compounds, acids, alkali, thermal treatment using pressing, heating, steam puffing, and decompressing. In some cases, pretreatment methods were combined. The sources and the method of extraction are the two major factors attributed to the production of cellulose from cotton wastes. However, there is still much more progress that is required. In recent years, methods of extraction have focused on different pretreatment methods, such as alkali and acid hydrolysis, including ultrasonication. Cotton production has increased over the years, resulting in the increase in cotton wastes that have been applied in different areas, such as environmental pollution control, soil amendment, reinforcements, and biomedicine. Studies have advanced on the increase in the crystallinity and yield of CNC produced from extracted cellulose from cotton wastes.

Recently, hydrolysis pretreatment has been combined with sonication to improve the cellulose content in waste cotton fibre. CNCs from cotton wastes have been used in the biopolymer matrix for reinforcements. The bio-composites have been used for packaging, building materials, and biomedical purposes. The chronological order of cellulose extraction from cotton wastes is presented in Table 3. Earlier studies of cellulose separation from cotton linter were reported by Talbot [118]. The separation was achieved by cooking cotton linter at a uniform temperature distribution in order to avoid the effect of poor liquid penetration. This approach was exhibited to remove the nanocellulose component in the cotton. Speakman [119] separated cellulose from cotton wool using sulphuric acid hydrolysis. The authors revealed that the filaments formed after pretreatment with sodium sulphide were attributed to the effect of formaldehyde. Masselli and Burford [120] extracted cellulose from cotton waste by bleaching using hypochlorite and peroxide. After then, sodium bisulphate was applied. The authors revealed that an increase in cellulose was achieved from the waste due to scouring. Kramar, Obradović [121] have attributed the increase in cellulose extraction for raw cotton silver waste to the influence of chemical pretreatment, bleaching, and scouring. Masselli [122] attributed the effect of scouring to an increase in the extracted cellulose for the removal of natural wax, pefins, alcohol, and impurities. The caustic kiering method was achieved using a mixture of sodium carbonate, caustic soda, and sodium silicate, resulting in the formation of the white fibre. The removal of natural wax from cotton was done by the addition of pine oil soap. Gallagher and Elliott [123] obtained cellulose from waste cotton cloths using alkaline scouring and peroxide bleaching of grey cotton cloth. The alkaline scouring was achieved via steaming of the cotton cloth impregnated with an alkaline medium. This resulted in the removal of the nanocellulose component of the cotton cloths. In 1980, enzymatic hydrolysis was used to obtain cellulose from cotton waste by compression-milling [124].

The energy requirement and enzymatic hydrolysis were affected by the milling time. The authors reported that the relationship between milling time, enzymatic hydrolysis, and the surface area of biomass determines the extent of crystallinity of cellulose materials. Maciel, Kolodziejski [125] separated cellulose from cotton linters by ball milling. The authors revealed that prolonged milling increased the cellulose chain due to the presence of carbon monomer units. Two years later, Bertran and Dale [126] extracted cellulose from cotton wastes by enzymatic hydrolysis. The authors attributed the low crystallinity from the cotton wastes to the susceptibility of the material to enzymatic attack. However, in a related study by Mokeev, Iljin [127], the effect of enzyme activities influenced the degradation and, by extension, the extraction of cellulose. Ludwig and Fengel [128] extracted cellulose from cotton linters by nitration using a mixture of nitric acid and sulphuric acid. The authors concluded that the significance of nitration was vital to the interaction between the cell wall, swelling capacity of the medium, and esterification on the surface of the cotton linters. Brooks and Moore [129] have extracted cellulose from cotton fabric waste by alkaline hydrogen peroxide bleaching. The authors emphasized that the hydroxyl anion was primarily responsible for the bleaching moiety in alkaline hydrogen peroxide.

Acid Hydrolysis has been reported for the separation of cellulose from cotton fabric waste. Elazzouzi-Hafraoui, Nishiyama [130] and Kuo, Lin [131] reported the extraction of cellulose from cotton wastes by enzyme saccharification. Wu, Zhang [132] extracted cellulose from cotton flax yarn waste using the alkali cooking method. The influence of an increase in temperature resulted in the water absorbency and bimolecular collision, which resulted in the increase in the extracted cellulose. The authors suggested that a cellulose macro-radical can be increased due to the effect of the collision. Bidgoli, Zamani [133] extracted cellulose from cotton and viscose waste (VW) by the dissolution of the wastes in water, followed by carboxymethylation. It was revealed that super adsorbent prepared from the extracted cellulose demonstrated effective water-binding capacity. Wang, Yao [4] have extracted cellulose by subjecting waste cotton cloth to alkali and bleaching treatments. Rough surface and disruption of the outer layer of the fibres and exposure of the fibril strands as a result of cracks in the inner structure of fibre were observed. Authors attributed it to the effect of bleaching and alkali treatment, causing the removal of major components, which include hemicellulose, waxes, and impurities. At present, the ease of cellulose extraction from cotton wastes has been improved by advanced technologies.

Ultrasonication is one method that has been used for the isolation of micro and macro fibrils and for the extraction of cellulose. A few studies have combined the process of hydrolysis and sonication for cellulose extraction. Pandi, Sonawane [134] stated that cellulose extracted from cotton wastes had been used in various applications, such as packaging and reinforcement for biomedical applications. This includes the production of CNCs of high crystallinity and thermal stability.

**Table 3:** Summary of the evolution of cellulose from cotton wastes.

Source	Method of Cellulose Extraction	Reference
Cotton linter	Cooking cotton linter, depolymerization	[118,135]
Cotton wool waste	Hydrolysis using concentrated sulphuric acid	[119]
Cotton Waste	Sodium bisulphite concentration	[120]
Cotton waste	Caustic kiering method, Geiger counter spectrometer	[122,136]
Cotton cloth waste	Kiering bleaching method	[123]
Cotton waste	Graft copolymerization	[137,138]
Cotton waste	Enzymatic hydrolysis by compressing milling	[124]
Cotton linters	Ball milling	[125]
Cotton waste	Enzymatic hydrolysis	[126]
Cotton linters	Nitration, organic solvent	[128,139]
Cotton waste	Strains of trichoderma and Aspergillus bleaching	[127]
Cotton fibre waste	Alkaline hydrogen peroxide bleaching	[129]
Cotton fabric waste	Hydrolysis using hydrochloric acid	[130]
Cotton waste	Ultrasonication, hydrolysis	[127,133,134]

### 3.2. Extraction of Nanocrystals from Cotton Wastes

The synthesis of cotton wastes on a nanoscale toward pollution management and environmental protection is drawing attention for diverse applications. The characterization of nanocrystals from cotton wastes is attracting significant interest. This can be attributed to its abundance of cellulose, environmentally-friendliness, high crystallinity, biodegradability, low toxicity, and high surface area, including its inherent optical properties and renewability potentials. The extraction of CNCs from cotton wastes can be achieved via chemical, mechanical, or enzymatic processes.

**Figure 10:**
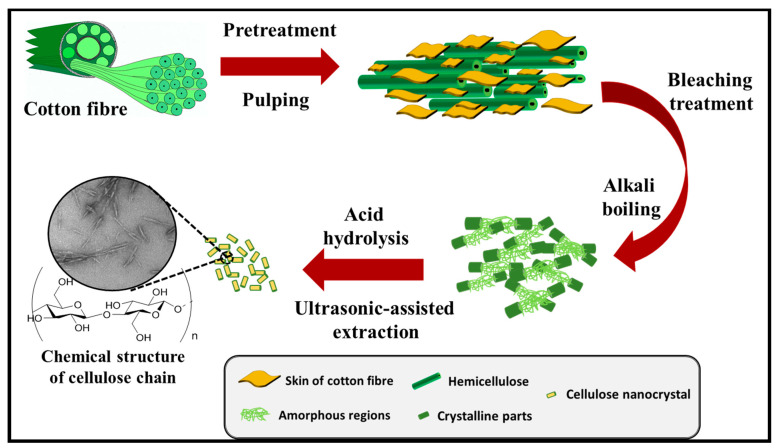
Schematic of nanocrystalline cellulose extraction from the cellulose chain using acid hydrolysis for the removal of the amorphous region [4].

The isolation of CNCs from semi-crystalline cellulose is usually achieved through a hydrolytic process whereby the amorphous phase undergoes digestion while, in the process of mechanical disintegration, the crystallites are released. Bahloul, Kassab [140] reported that the characteristics of CNC are affected by the source of cellulose, hydrolysis condition, and reaction temperature. References [141,142] reported that CNCs could be extracted from sources such as cotton stalks, cotton linters, and cotton slivers. Khalil, Davoudpour [142] have reported that extraction of CNC is mostly achieved using mineral acids such as hydrochloric, phosphoric, and sulfuric acids. The extraction of the cellulose chain by the removal of the amorphous region from cellulose-composed materials is shown in Figure 10.

Maciel, de Carvalho Benini [143] have reported the extraction of CNC from cotton waste fibres by hydrolysis. It was revealed that the chemical treatment was responsible for the decrease in the amounts of hemicellulose and total lignin (soluble + insoluble), including the extractives and ash. As a result, the relative amount of cellulose was found to increase for bleached fibres compared to the untreated ones. Kalia, Thakur [144] stated that the effect of chemical treatments did not only decrease the amorphous components but also were responsible for the colour changes from brown to white. The findings revealed that cotton wastes provided a good source of cellulose and for the extraction of nanocellulose. Pandi, Sonawane [134] extracted CNC from cotton wastes by bleaching, hydrolysis, and sonication pretreatment methods. High stability and crystallinity of CNC were found higher than the raw material. The combined effect of hydrolysis and sonication can possibly isolate more CNC from cotton. Thambiraj and Shankaran [34] have isolated CNC from cotton wastes by hydrolysis using H_2_SO_4_. The produced CNC demonstrated high crystalline, biocompatible, and sustainable behaviour. The type of acid used for extracting CNCs can affect the characteristics of the CNC. When sulfuric acid was used, sulphate groups from the acid esterifies the free hydroxyl groups on the surface of the CNC. Conversely, CNCs hydrolyzed by HCl have relatively low concentrations of strong and weak acid groups bound on the surface that allow the crystals to aggregate and flocculate due to van der Waals attraction in aqueous solutions.

**Figure 11:**
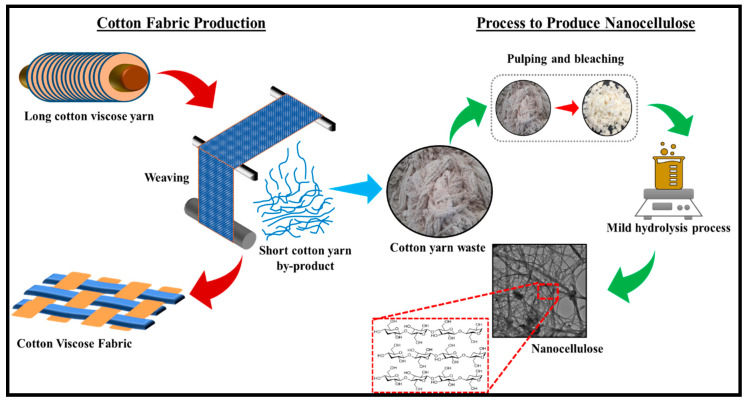
Viscose yarn waste production and one-step extraction of NC from VW [149].

Wang et al. (2019) extracted CNC from cotton wastes by alkali treatment, which is followed by acid hydrolysis. The extracted CNC demonstrated smooth and flat surface morphology, including a high aspect ratio. CNC exhibiting a high aspect ratio have the reinforcing potentials in composites due to good mechanical properties. Reference [145] stated that the efficiency of CNC for reinforcement applications is influenced by the crystallinity and the aspect ratio. This implies that cotton-derived CNC can serve as an effective reinforcement material for various applications. According to Kassab, Kassem [146], a high aspect ratio CNC provides a reinforcing effect due to the good physical and mechanical properties. Hemmati, Jafari [147] reported the extraction of CNC from cotton linters by alkali treatment using NaOH. High crystallinity of CNC was achieved. Although, the thermal stability was lower than the raw material. This problem was caused by an increase in the hydrolysis concentration, leading to fragmentation of cellulose particles. Culsum, Melinda [148] extracted CNC from denim wastes using alkali treatment and ammonium persulfate (APS) oxidation at 60 °C within 5 to 15 h. Zhong, Dhandapani [141] have synthesized CNC from indigo-dyed denim fabrics by bleaching 2 m long cotton fabrics using contra selector mill, and then hydrolysis using 64 wt% of H_2_SO_4_ at 45 °C for 1 h. Higher crystallinity of CNC was achieved. This was attributed to the susceptibility of the amorphous region as a result of the effect of hydrolysis, which influenced the removal of amorphous cellulose. Reference [149] extracted CNC from viscose yarn via a facile one-step process (Figure 11). Higher crystallinity of CNC was achieved. This was attributed to the susceptibility of amorphous region as a result of the effect of hydrolysis, which influenced the removal of amorphous cellulose.

The extraction of CNC from cotton wastes, pretreatment methods, and process conditions, including their strength and limitation from the previous studies, is presented in Table 4.

The extraction of CNC from cotton wastes is achieved at a low cost. The wastes are harnessed into value added products with promising potentials for a wide range of application. The functional properties of cotton wastes as a precursor for the extraction of CNCs depend on the extent of its crystallinity. The crystallinity of a material is a function of the hydrolysis reaction time, which is determined by the crystalline index [151]. Ilyas, Sapuan [152] stated that the formation mechanism of the nanostructure is based on the extent of susceptibility of the amorphous phase to acid hydrolysis in comparison with the crystalline phases. The extent of the removal of the amorphous phase relative to the amorphous region is affected by the hydrolytic time in view of extraction of cellulose from cotton wastes. Zhong, Dhandapani [141] and other studies have reported extraction of CNCs from cellulose derived from cotton wastes (Table 4). The functionality and efficiency of the produced CNCs were determined from the yield and crystallinity index. The result of a comparative study of the crystallinity and yield of CNC extracted from cotton wastes is presented in Table 5.

The findings of the crystallinity indexes in Table 5 is discussed on the basis of the extraction process in Table 4, which is relative to the source of the waste and the pretreatment methods. Maciel, de Carvalho Benini [143] reported a high crystallinity index and yield from the CNC processed from cotton waste. The high crystallinity index was attributed to the effective removal of amorphous compounds before hydrolysis. Reference [148] achieved higher crystallinity similar to the value obtained for CNC derived denim wastes from cotton fibres [141]. Low hydrolytic time of extraction of CNC from indigo denim fabrics produced CNC of high crystallinity and thermal stability. The significance of high crystallinity at low hydrolytic time indicated that the highly crystalline CNC produced could be attributed to the effect of bleaching since sulfuric acid hydrolysis could not degrade indigo dyes. The inability of hydrolysis by sulphuric acid to degrade indigo dyes was attributed to the poor yield of CNC obtained from cotton wastes [141]. Morais, de Freitas Rosa [156] have extracted nanocellulose from cotton linters. The authors revealed that the CNC have prospects for the synthesis of hydrophilic nanocomposites. The linter have potentials for the formation of CNC. Hemmati, Jafari [147] achieved high crystallinity and yield of CNC from cotton linters. The CNC obtained have high water holding capacity compared to the raw cellulose. The water holding capacity is attributed to the larger pore width size of the extracted CNC. The amount of amorphous region is expressly removed at the expense of crystalline cellulose as a result of the removal of hydrogen bonding in the network structure. Therefore, the extracted CNC have higher water sorption capacity as a result of the increased width of the CNC. The high crystallinity can be attributed to the preferential hydrolysis of the cellulose amorphous regions, which degraded during hydrolysis of sulphuric acid into soluble products [157].

Furthermore, Wang, Yao [4] reported that the crystallinity of CNC derived from cotton cloth waste was not too high. Low thermal stability of CNC was achieved when compared to the raw material. This is because the crystalline domains were found to be intact due to the arrangement of a highly ordered molecular chain that resisted acid penetration. Higher stability of suspension in the CNC produced is attributed to the high crystallinity of the CNC. Although there was evidence of poor thermal stability in some of the produced CNC, this can be attributed to the pretreatment methods and the nature of the surface of the wastes. In addition, the hydrolysis reaction affected the amorphous and crystalline region. In a related study, Reference [158] used a mechanical, high-pressure homogenizer for the extraction of CNC from the cotton fibre after pretreatment using sulphuric acid hydrolysis. The concentrations of sulfuric acid, hydrolysis time, and homogenizer speed were the optimum conditions that resulted in CNC of high crystallinity and thermal stability. High crystallinity and good thermal stability of the CNC could be attributed to the mercerization process. Authors suggested that the mercerization process increased the size of the crystallites.

Thambiraj and Shankaran [34] synthesized cellulose microcrystals (CMCs) from industrial waste cotton by hydrolysis to produce CNC. The prepared CNCs from waste cotton was performed via a two-step procedure. First, CMCs were extracted from the cotton wastes by the removal of pectin including hemicellulose by alkaline hydrolysis and then lignin in an acidic condition. The effect of alkali treatment in the first stage was to remove and hydrolyze the hemicellulose contents including ash, minerals, and other forms of impurities. The impact of alkaline hydrolysis influences the morphology of the produced material. The result revealed that cotton fibre treated at 70 °C have a smooth and well-aligned surface. The removal of the amorphous region was achieved in the second process by acid hydrolysis in order to obtain a crystalline region of CNCs. The result from the findings indicated that the isolated CNCs demonstrated high crystallinity, excellent optical properties, and thermal stability. It is suggested that the improved properties of the isolated CNC is attributed to a high aspect ratio. This is because a high aspect ratio plays a prominent role in increasing the path length of permanent molecules. As a result, the barrier properties are improved. This is shown in the improved mechanical properties of the extracted CNC.

**Figure 12:**
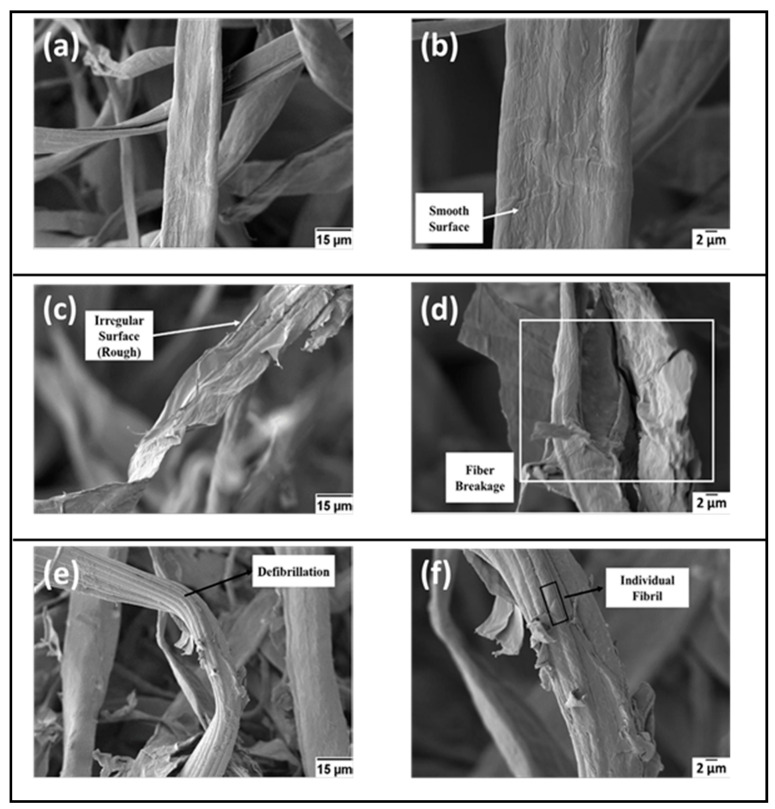
SEM Micrograph of (**a**,**b**) untreated, (**c**,**d**) alkali-treated, and (**e**,**f**) bleached waste cotton fibres [143].

Previous studies have indicated the significance of the hydrolysis reaction in the extraction of CNC. The effect of chemical hydrolysis in the removal of an amorphous region can be observed in the difference in the morphology of treated and untreated cotton waste fibres, as illustrated in Figure 12.

**Figure 13:**
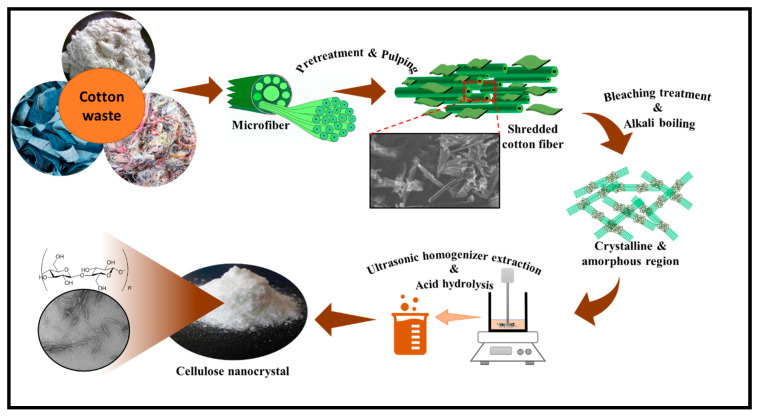
A summary of extraction methods of CNC derived from cotton wastes.

In Figure 12a,b, the morphology of the untreated fibres indicated a smooth surface with predominance of fibre bundles. The effect of alkali hydrolysis affected the morphology of the fibre in Figure 12c,d. Very rough and irregular surfaces that include a breakage of fibre can be observed. This is attributed to the removal of the superficial layer, which was formed by wax [159]. After the bleaching of fibre, more expressive removal of amorphous contents was achieved. The removal of the amorphous region including hemicellulose and lignin resulted in the production of fibrils via fibrillation (Figure 12e,f). A well-structured superfine fibril of cotton fibers consisting of high cellulose content is an ideal precursor for the extraction of CNC. An observable trend of residue weight decreases as a result of the progression of chemical treatment noticed, suggesting that the effect of alkali and bleaching treatment on the cotton fibres effectively removed the amorphous content and other impurities. This process is very significant due to the effect of the components on the crystallinity and thermal stability of the extracted CNC. A summary of the extraction processes of CNCs derived from cotton wastes is presented in Figure 13.

Chemical pretreatment is the most used extraction method for CNC from cotton wastes precursor. However, the techniques applied to extract CNC depends on the chemical composition of the precursor and the pretreatment process conditions of extraction. The extraction procedures involve four major steps, which are washing to remove compounds from cotton wastes, pulping of cotton wastes, then bleaching, and, finally, hydrolysis. The extraction of CNC from cotton wastes using chemical pretreatment mostly applied sulfuric acid hydrolysis to achieve CNC of high crystallinity. Bleaching and sulfuric acid hydrolysis is a highly efficient process of producing CNC of high purity and a high aspect ratio, which can suitably be utilized as reinforcement material in a polymer composite. A combined acid hydrolysis using H_2_SO_4_ and HCl produced a very dense, flat, and smooth surface of CNC with good thermal stability. The extracted CNC when used as a reinforcement agent exhibited good surface area, high crystallinity, tensile strength, and modulus of elasticity. However, the CNCs were not well distributed in the polymer matrix at a high concentration. This could be improved using ultrasonic assisted hydrolysis. This process could produce CNC of high crystallinity, a high aspect ratio, and higher thermal stability. However, the dispersibilty for CNCs is caused by hydrolysis of HCl. This problem could be improved by the electrostatic repulsion of sulphate ions during hydrolysis with a mixed acid. The combined effect of sonication and hydrolysis using ultrasonic assisted H_2_SO_4_ hydrolysis could enhance the structural and thermal properties of CNC extracted from cotton wastes. This included increased crystallinity due to a combined effect. Enzymatic hydrolysis and sonication could produce high crystallinity CNC from cotton wastes. Low power and short sonication could disrupt the weak linkages of the hydrolyzed cotton for CNC extraction. Lastly, sulfuric acid hydrolysis assisted high pressure of a mechanical homogenizer that could increase crystallinity and a high aspect ratio of CNC from cotton wastes, which has shown to produce composites of high tensile strength.

### 3.3. Advanced Applications of Cotton Derived CNC

With recent advances in extraction of CNC from cotton wastes using different isolation techniques, there is remarkable interest in advancing its potentials in various applications, which will be a significant benefit in nanotechnology. The utilization of the intrinsic properties of derived CNC from cotton wastes have prospects in advancing applications in areas such as biomedical and packaging. The design of nanocomposite films using cotton-based CNC as reinforcement to enhance films’ properties have attracted significant attention in recent years to improve properties or as a replacement for existing applications.

#### 3.3.1. Biomedical Engineering

The potential of CNC has been explored for biomedical application, such as anti-cancer activity, drug delivery, and tissue engineering in previous studies [160]. Traditionally, carbon nanotubes have been utilized for drug and gene delivery due to their effective penetrability and their encapsulation efficiency. Reference [161] reported the potentials of cotton-derived CNC as replacement of carbon nanotubes for drug delivery. The authors investigated advanced application of cotton-based CNC functionalized with disulfide bond-linked poly (2-(dimethylamino) ethyl methacrylate. The condensation ability of the gene including reduction sensitivity, cytotoxicity, gene transfection, and in vivo anti-tumor activities were investigated. It was revealed that the cotton-based CNC demonstrated very effective transfection efficiencies and low cyto-toxicities. The toxicity of CNCs through pathways such as inhalation into the lungs and cellular uptake is a critical property that determines the utility of the nanoparticles in the biomedical sector [162]. Reference [163] fabricated a gelatin composite film using cotton as reinforcement for a wound dressing application. The developed bio-composite film exhibited homogenous dispersion of the CNC within the gelatin matrix including a strong interfacial interaction between the matrix and the reinforcement. The evidence of excellent biocompatibility indicated improved mechanical properties of the composite film. Authors found no cytotoxicity from the in vitro analysis, which suggested the significance of the reinforcement in advanced application of the composite film for wound dressing. Reference [117] compared the interaction of CNC derived from cotton with CNC extracted from tunicate using a multicellular in vitro model of the epithelial airway barrier after aerosol exposure. Authors reported that an increase in the doses of short cotton CNCs were readily cleared from the surface by internalization within 24 h after exposure when compared to tunicate CNC. However, both extracted CNC exhibited no translocation and no clearance pathway. The fibres alone were able to pass through the insert membrane. This gives a valuable insight when considering the long-term effect and consequences of CNC exposure to humans. However, CNC is found to be non-cytotoxic. Further in-depth studies are required, such as immunogenicity and blood compatibility, which are currently lacking in the literature. A non-cytotoxic CNC could interact with biomolecules and evoke other responses in the biological systems. Therefore, advanced studies are required to further explore the potentials of cotton-based CNC as a replacement for carbon nanotubes for medical applications.

#### 3.3.2. Packaging Applications

The reinforcement potentials of CNCs have often been explored for the improvement of mechanical properties of composites for packaging applications. Reference [164] compared the potentialities of CNC isolated from cotton linters under two different processing conditions as coating for Poly (ethylene terephthalate) films. The pretreatment processes were sulphuric acid hydrolysis and ammonium persulfate (APS) oxidation. It was revealed that CNC isolated using APS oxidation exhibited a higher charge density, higher crystallinity, and higher transparency of the coating compared to sulphuric acid that extracted CNC. Higher oxygen barrier was exhibited by the sulphuric acid-treated CNC. This indicated that the potential surface modification or grafting is required for improved properties of the APS-treated CNC. Although both CNCs demonstrated a lower oxygen permeability coefficient compared to commonly used synthetic resin for packaging. There is a need for coating the layer of the APS-treated CNC to enhance the final properties of the packaging application and as an alternative to other food packaging materials. Reference [165] investigated the mechanical, optical, and anti-barrier properties of polyethylene terephthalate (PET), oriented polypropylene (OPP), oriented polyamide (OPA), and cellophane films coated using CNC extracted from cotton linters. The authors reported that CNC-coated PET and OPA exhibited the best performance when compared to other coating films. The less effective performance of OPP was attributed to the weak adhesion interaction between CNC coating and the OPP surface, which resulted in the removal of CNC from the substrate during dynamic measurements. A well-dispersed CNC coating on the substrate is likely to improve the surface property of the OPP for the packaging application. Jiang et al., 2021, extracted CNC from cotton fibre and the extracted CNC was applied as reinforcement for composite films. The addition of the CNC as filler improved the tensile strength of the fabricated film. Oun and Rhim, 2015 reported that cotton linters have been used as a precursor for the extraction of CNC and investigated its effect on films’ property for the production of the composite. The improved tensile strength of the film including water vapour barrier properties indicated the effectiveness of the CNC as filler for the reinforcement of the composite. Huang et al., 2020, reported the extraction of CNC from cotton wastes and was applied for the reinforcement of soy protein film. The incorporated CNC improved the tensile properties, Young modulus, and water vapour barrier property of the composite film. This implies that cotton wastes can be successfully applied as an effective alternative in blended composites and can complement other fibres for the improvement of mechanical and thermal properties for packaging applications.

## 4. Challenges of Cotton Derived CNC and Prospects

Cotton wastes is the most generated wastes during textile production. The ineffective disposal mechanism of the wastes causes environmental problems. The valorization of cotton wastes into functional materials is essential, but its sustainability and the potentials of extracted cellulose from the waste streams have not been fully explored even though they have been utilized for some geotechnical, environmental, and biomedical applications. A cotton fiber–biopolymer blend for functional and advanced applications such as biomedical, tissue engineering, etc. still requires further research. Issues of biocompatibility and toxicity of the cotton fibre are still pending. The extraction of CNC from cotton wastes have been widely reported in the literature. However, there still exists a drawback that could limit their applications. The pretreatment strategies can suffer variation in terms of process conditions, which could result in a distortion in the crystallinity of the extracted cellulose. Chemical pretreatment is considered the most efficient process. In some instances, an increase of acid concentration affects the crystalline region of the CNC. The use of ultrasonic techniques for the extraction of CNC from cotton wastes is attracting attention in the area of sonochemistry.

**Figure 14:**
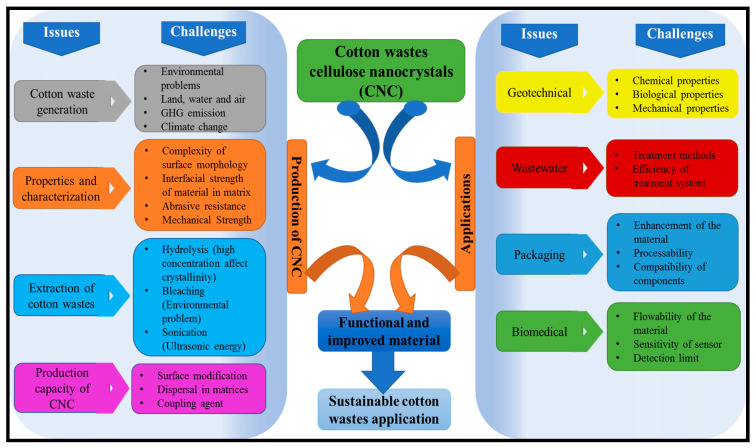
Issue challenges of extraction methods for the production of CNC.

In an ultrasonic technique, the exchange of ultrasonic energy to the cellulose chain requires energy for the breaking down of cellulose into nanofibers. Sonication depends on cavitation, whereby the ultrasound energy may likely cause shear-in resulting in breakage of intra-fibrillar bonds due to scission. On a commercial scale, ultrasonication is effective for the extraction of nanofibers from the original, natural fibre by altering the duration and power of the ultrasound. The diameter of the nanocrystal can be increased in the process. Many studies have combined pretreatment methods, such as hydrolysis, alkali treatment, and sonication, to reduce production cost. An increase in the acid hydrolysis affects the crystalline region in some cotton wastes. Therefore, more studies should focus on the best approach to minimize the effect of hydrolysis for an optimal benefit. Mass production of CNCs from cotton wastes have rarely been explored on industrial level even though there are promising potentials for various industrial applications, such as biocompatibility, biodegradability, and high thermal conductivity. The issues and challenges that affect the production of CNC from cotton wastes documented in this review is illustrated in Figure 14.

Furthermore, cotton wastes as a precursor for activated carbon have demonstrated high adsorption capacity for the removal of recalcitrant pollutants for dyes and heavy metals including organic pollutants in solution. However, the sorption capacity of the wastes adsorbent depends on the operational conditions of the treatment system and the surface structure of the wastes adsorbent. Surface modification of cotton wastes will improve the extent of attachment of pollutants on the active sites of the adsorbent. The challenge of improvement of reinforced matrix interface using cotton fibre as reinforcement in composites is still a subject of intensive research. According to Claramunt, Ventura [166], the reinforcement of the composite with natural fibres are usually dispersed randomly on the matrix in textile structures, such as woven or nonwoven fabrics. This is because short fibres are used for reinforcements. Even though the flexural strength and ductility of the composite are improved by the randomly dispersed fibres, the improvement is limited due to the short length of the fibres. In some other cases, nonwoven cotton fabrics are rarely applied in cement composites due to the problem of infiltration of the cement matrix as a result of the closed structures. These challenges need to be eliminated through comprehensive research. Furthermore, the mechanical properties depend on the fibre type, orientation, and degree of entanglement. In view of producing cotton fibre-reinforced cement composite for structural application, studies on cotton fibres in a cement matrix with a high degree of fibre entanglement are still very limited. However, more research needs to be done for improved properties of cotton fibres for reinforcement of composites. Cellulose derived from cotton wastes have demonstrated remarkable potential for packaging and biomedical applications.

The possibility of combining biopolymers and cellulose from cotton wastes to obtain a biodegradable plastic can be very useful for packaging applications. The effect of cellulose as filler can significantly impact the mechanical properties of the biopolymer. Masmoudi, Bessadok [167] reported that an increase in cellulose in the biopolymer increases the elongation rate. There was minimal elongation at break as a result of strong adhesion at a saturation between the filler and the matrix. This can be attributed to the increasing forces of the interaction of density and interfacial tension. To a large extent, through innovative ideas, cotton wastes have been synthesized to nanocellulose materials and have found wide application in various areas of nanotechnology, ranging from non-food packaging, bio-nanocomposites, environmental, and biomedical applications. The availability of the technologies for producing CNCs from cotton wastes with enhanced properties having stable, fluid aqueous dispersion, abundant surface functional groups, and a strong interaction with a polymer matrix are subject to future development, considering its socio and economic benefits.

## 5. Propositions for Future Research on Nanocellulose Crystals from Cotton Wastes

The development of CNCs from cotton wastes have demonstrated vast potentials for various application in our earlier discussions. For the advancement of nanotechnology, enhancement by chemical modification, adding compatibilizers to improve CNC is significant since hydrophilic behaviour of CNC inhibits mixing and dispersal in hydrophobic matrices. PLA obtained from agricultural sources combined with nanocellulose produced cellulose extracted from cotton wastes as reinforcement have the potential to create a functional CNC. The new biomaterials have the prospect of creating new areas of applications in construction and automotive engineering including a sustainable water treatment system, biomedicine, and food packaging. However, the challenge of the dispersal of the reinforcement in the polymer matrix without causing degradation of the biopolymer may limit the application. This limitation can be improved through the nanofibers-matrix compatibility by an interaction or by adopting suitable processing techniques. The technique to be adopted to manufacture a biopolymer/cotton-derived nanocellulose composite should be on the basis of the desired application. This includes the physico-chemical characteristics and the thermal stability of the nanocellulose. For example, the use of nanocellulose as an additive for reinforcement of the biopolymer must have effective binding potential, high molecular strength, and functional surface chemistry that allows reactivity. Cellulose nanocomposites are fibrous in nature with effective mechanical properties including their biocompatibility. They are potentially suitable as components for improved membranes for water treatment systems, conductive thin films, wound dressing, and paper barriers. The incorporation of CNC within the polymer matrix alters the membrane properties. More research attention should focus on the enhancement of the tensile strength from the added CNC and the hydrophilic behaviour, permeability, and resistance to biofouling of the produced bio-nanomaterial.

## 6. Conclusions

Cotton wastes have been effectively applied due to the high adsorption capacity of the precursor for the removal of dyes and heavy metals from solution. The functional properties of cotton wastes have been utilized for the production of lightweight composite-building materials. Cotton wastes have found application as filler for reinforced structures for geotechnical applications for slope amendments as well as reinforcement of soils and composites with effective binding properties. The cellulosic properties of cotton wastes with other pozolanic materials could be an effective alternative binder to improved flexural strength and physical properties of materials for packaging. Cotton fibres have also been utilized as effective reinforcement materials for packaging, antimicrobial materials, and for the fabrication of scaffolds for tissue-engineered applications. The extraction of CNCs from cotton wastes offer remarkable prospects to produce functional materials for reinforcement applications of composites. The processing of cotton wastes for the extraction of CNCs holds promising potentials for advanced application due to the high crystallinity and thermal stability. Several novel extraction methods including a combination of processes have been reported. Acid hydrolysis using sulphuric acid remains the most used pretreatment method. Cotton gin motes and cotton gin wastes could extract CNCs of high thermal stability and high crystallinity using sulfuric acid hydrolysis. CNCs of high crystallinity could be extracted from cotton wastes by alkali treatment and acid hydrolysis. A combined process of bleaching, acid hydrolysis, and sonication could extract CNC of high thermal conductivity from cotton wastes compared to the raw material. The crystallinity of cotton wastes derived CNC that could increase by 81.23% and the yield by 45%. Reduction in hydrolysis time and enhanced yield of CNC could be achieved by combined extraction methods. Physical and chemical modification of cellulosic sources derived cotton wastes significant for improved functional materials for improved packaging and biomedical applications through improved bonding of the fibre and the matrix of bionanomaterial products. Cotton wastes-extracted CNC have been incorporated as a reinforcement agent in composite films with improved compatibility, homogenous dispersion, and effective fiber-matrix interaction for packaging. CNC derived from cotton wastes have proven to exhibit a non-cytotoxic effect and could be applied as a replacement for carbon nanotubes for biomedical applications. The high crystallinity of CNCs derived from cotton wastes and their properties including high tensile strength and high-water holding capacity create prospects for the production of CNC aerogels/hydrogels for drug delivery, building insulations, and oil and water separation applications. Previous researchers have explored the ideas of producing CNCs. However, effort toward advancing the commercialization potential remains a challenge. Future works to improve on the dispersibility of CNC in the polymer matrix as reinforcements, including the potentials of CNCs from cotton wastes to produce novel bionanomaterials, need to be explored.

## Figures and Tables

**Table 4:** Cotton wastes and method of extraction of CNC.

Sources	Pretreatment Methods	Process Condition	Average Size of CNC	Advantages	Disadvantages	Reference
Waste cotton cloth	Alkaline treatment and acid hydrolysis	Alkali treatment 10% (wt%) of NaOH for 2 h Hydrolysis: H_2_SO_4_ and HCl at 55 °C for 7 h using ultrasonic waves	Length 28 to 470 nm Diameter: 35 nm	High crystallinity of CNC.	Low thermal stability compared to raw material	[4]
Cotton wastes	Acid hydrolysis	Hydrolysis: 60% H_2_SO_4_ at 50 °C for 8 h	Diameter: 6.5 nm Length: 180 ± 60 nm	Unique fluorescence properties for bioimaging and biosensing applications	Energy time consumption to determine fluorescence property	[34]
Waste cotton cloth	Alkali treatment and acid hydrolysis	Alkali treatment NaOH at 70 °C for 2 h Hydrolysis: H_2_SO_4_, HCl, at 65 °C for 5 h	Length: 38 nm to 424 nm Diameter: 2 to 17 nm	Smooth and dense surface with high crystallinity	Excess CNC will inhibit the formation of the transparent film in composite	[150]
Cotton waste	Bleaching, acid hydrolysis, and sonication	Bleaching at 60 °C for 4 h using NaOCl Hydrolysis: H_2_SO_4_ (30–50%) for 4 h Sonication: Ultrasound probe sonication for 45 min	Length: 20–100 nm Diameter: 10–50 nm	High thermal stability than the raw material	The combined effect of hydrolysis and ultrasound treatment is expensive	[134]
Cotton wastes	Alkali treatment, bleaching, and acid hydrolysis	Alkali treatment: NaOH solution for 1 h at 70 °C. Bleaching: Added H_2_O_2_ NaOH for 1 h at 50 °C Hydrolysis: H_2_SO_4_ at 50 °C. Pulp: Solution of 1:20 (g/mL)	Length: 105–5880 nm Diameter 23.8 ± 5.6	Effective removal of amorphous compounds before hydrolysis	Increase in reaction time not feasible at 15 min due to an increase in processing cost	[143]
Indigo Denim fabrics	Bleaching and acid hydrolysis	Bleaching cotton fabrics using cotton selection mill Hydrolysis: H_2_SO_4_ at 45 °C for 1 h	Length: 197 Diameter: 7	High crystallinity and thermal stability	Sulphuric acid hydrolysis could not degrade indigo dyes	[141]

**Table 5:** Comparative study of material properties of cellulose nanocrystals obtained from cotton wastes.

Source	Size Width (nm)	Length (nm)	Crystallinity Index (%)	Yield (%)	Reference
Cotton waste	221	20–100	81.23	45	[134]
Denim waste	80–120	76.14 ± 8.56	86	24.14	[148]
Cotton fibre from denim fabrics	11.9 ± 6.7	127.7 ± 43.8	86.4	39.6	[141]
Cotton gin motes and cotton gin wastes	78–247	100–300	78	29.3–48.6	[153]
Cotton waste	-	105–5880	75–81	80–89	[143]
Cotton linter	133	229 ± 97	82	59	[147]
Cotton waste	10 ± 1	180 ± 60	-	45	[34]
Waste cotton cloth	-	28–470	55.76 ± 7.82	46.7 ± 1.8	[150]
Cotton waste	40–90	70–200	82.80	25.21	[154]
Cotton waste	-	76–159	79.	30–35	[155]

## Data Availability

Not applicable.
